# Late Presentation of Posterior Urethral Valve in a Four-Year-Old Child With Subtle Voiding Symptoms: A Case Report

**DOI:** 10.7759/cureus.77463

**Published:** 2025-01-15

**Authors:** Hany M Elkordy, Mohamed Elkordi, Greta Peciulyte

**Affiliations:** 1 Urology, Prime Healthcare Group, Dubai, ARE; 2 Urology, NMC Royal Hospital, Abu Dhabi, ARE; 3 Urology, Harley Urology LTD, London, GBR

**Keywords:** bladder function, endoscopic laser ablation, lower urinary tract obstruction, posterior urethral valve, urethrocystoscopy, voiding cystourethrography, voiding urinary symptoms

## Abstract

A four-year-old male child following an attack of acute pharyngitis was referred to the urology department as having mild voiding difficulty, where posterior urethral valve (PUV) was incidentally diagnosed. Urethrocystoscopy confirmed Type I PUV, which was successfully managed with transurethral laser ablation. This case underscores the importance of evaluating subtle pediatric urinary symptoms to prevent renal complications. Advances in imaging, surgical techniques, and monitoring strategies are critical for effective management. Early diagnosis and timely intervention remain essential to preserve renal and bladder function, emphasizing the need for vigilance in assessing pediatric urinary complaints.

## Introduction

Posterior urethral valve (PUV) is the most common cause of congenital lower urinary tract obstruction (LUTO) in male patients, accounting for approximately 60% of cases. If left untreated, it can lead to significant renal and bladder dysfunction and may even become life-threatening. The incidence of PUV is estimated to be one in 7,000-8,000 live births [1,2].

According to Hugh Young's classification, PUV is categorized into three types, with only Type I and Type III considered obstructive. Type I, the most common form (90%-95% of cases), involves membranous folds extending from the verumontanum to the posterior urethra. Type II, which is rare, involves folds extending from the bladder neck to the verumontanum. Type III presents as a distal membranous diaphragm at the posterior urethra.

Recent studies have highlighted genetic predispositions linked to PUV, including associations with TBX5 and PTK7 genes, emphasizing the potential role of genome-wide association studies (GWAS) in understanding its pathogenesis [3]. Advances in prenatal ultrasonography have significantly improved the detection rates of LUTO, allowing for earlier diagnosis and intervention [4,5]. For postnatal assessment of PUV, a voiding cystourethrogram (VCUG) is recommended, including lateral views of the urethra during the voiding phase without a catheter in situ [6].

Management strategies for PUV range from prenatal interventions in specialized centers to postnatal approaches, including bladder drainage, vesicostomy, endoscopic valve ablation, and clean intermittent catheterization, and in severe cases, higher urinary diversion procedures may be needed [5,7].

## Case presentation

A four-year-old boy was referred to the urology clinic with mild voiding symptoms, including a weak urine stream and straining to void, which had started two weeks prior to presentation. This followed an episode of acute pharyngitis. The parents reported that he had rarely experienced similar symptoms in the past and had otherwise maintained full bladder control. He had no prior significant medical or surgical history. Physical examination revealed normal external genitalia and unremarkable abdominal findings.

**Figure 1:**
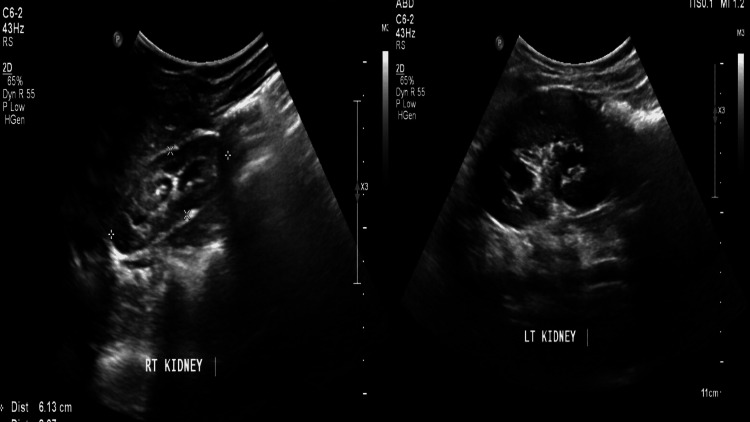
Ultrasound exam of the kidneys, ureters, and bladder (US-KUB) demonstrated mild-to-moderate bilateral hydronephrosis.

Laboratory findings revealed a serum creatinine level of 0.55 mg/dL, blood urea of 36 mg/dL, and normal complete blood count and electrolyte profile. Urine analysis and culture showed no bacterial growth. Ultrasound exam of the kidneys, ureters, and bladder (US-KUB) demonstrated mild-to-moderate bilateral hydronephrosis, thickened bladder wall, and dilated distal ureters, suggesting infravesical obstruction or reflux (Figure 1).

**Figure 2:**
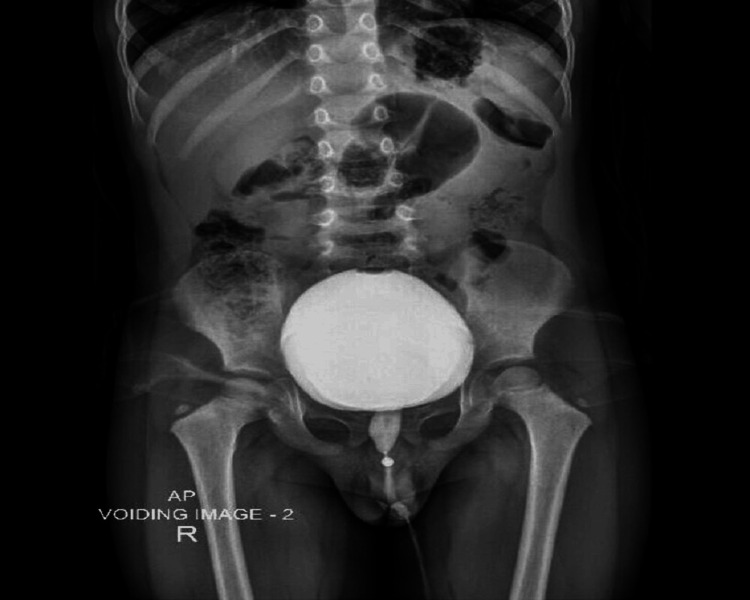
A voiding cystourethrogram (VCUG) revealed a dilated posterior urethra without evidence of vesicoureteral reflux, consistent with a posterior urethral valve initial diagnosis.

A VCUG revealed a dilated posterior urethra without evidence of vesicoureteral reflux, consistent with a PUV initial diagnosis (Figure 2).

**Figure 3:**
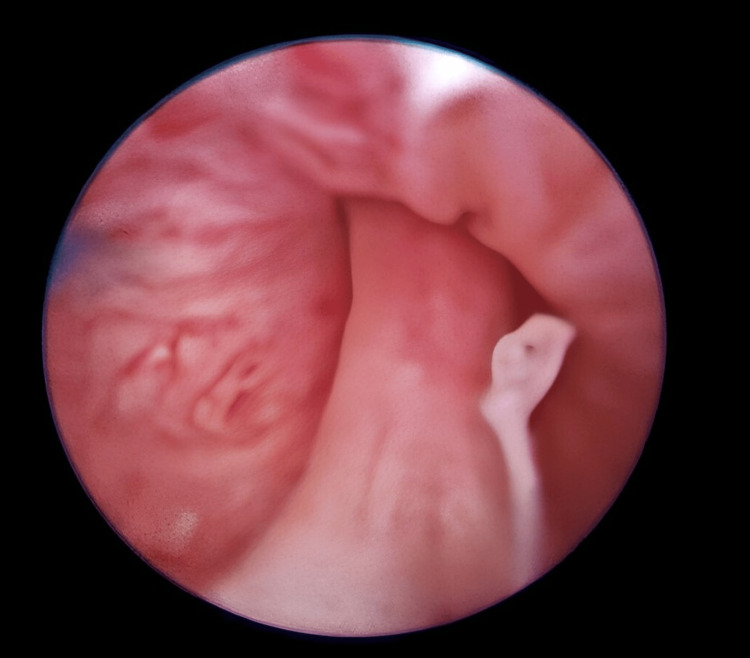
Urethrocystoscopy confirmed the diagnosis of Type I posterior urethral valve (PUV). A successful laser ablation was performed.

After obtaining informed consent, we proceeded with diagnostic urethrocystoscopy under general anesthesia, and it confirmed Type I PUV, which was subsequently treated with laser ablation (Figure 3).

**Figure 4:**
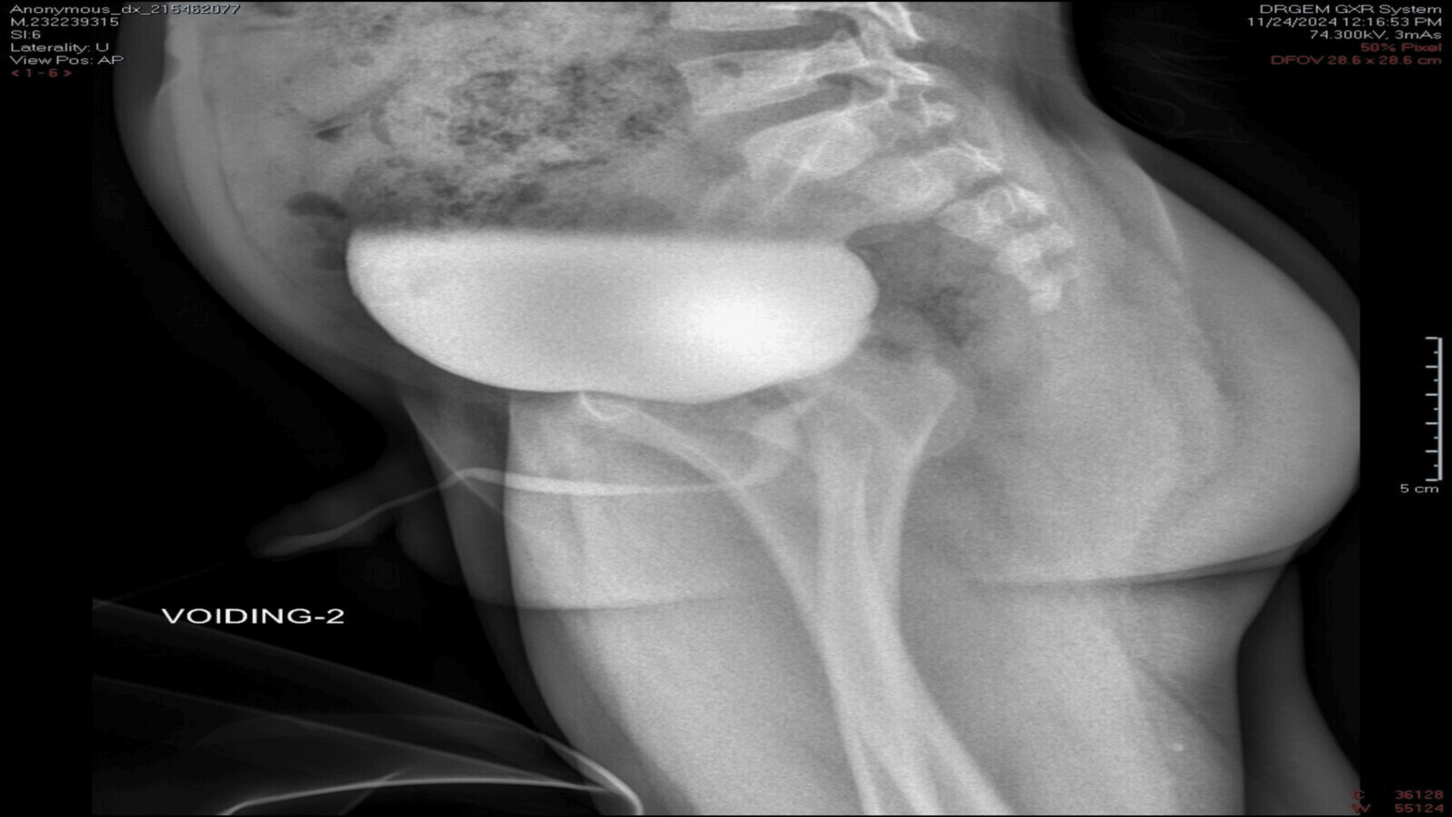
A voiding cystourethrogram (VCUG) showed resolution of the previously noted posterior urethral dilatation.

Endoscopic laser ablation of the PUV was performed successfully at the five and seven o’clock positions using a Holmium YAG laser at a setting of 1 J to 15-20 Hz. Cystoscopy revealed mild urinary bladder trabeculations. Both ureteric orifices were identified with no visible mass or diverticulum. The child was catheterized with Foley’s catheter for only 24 hours. Postcatheter removal, the child voided freely without complications. Follow-up results nine months later revealed normal renal function tests; VCUG showed resolution of the previously noted posterior urethral dilatation with no apparent urethral filling defects (Figure 4).

**Figure 5:**
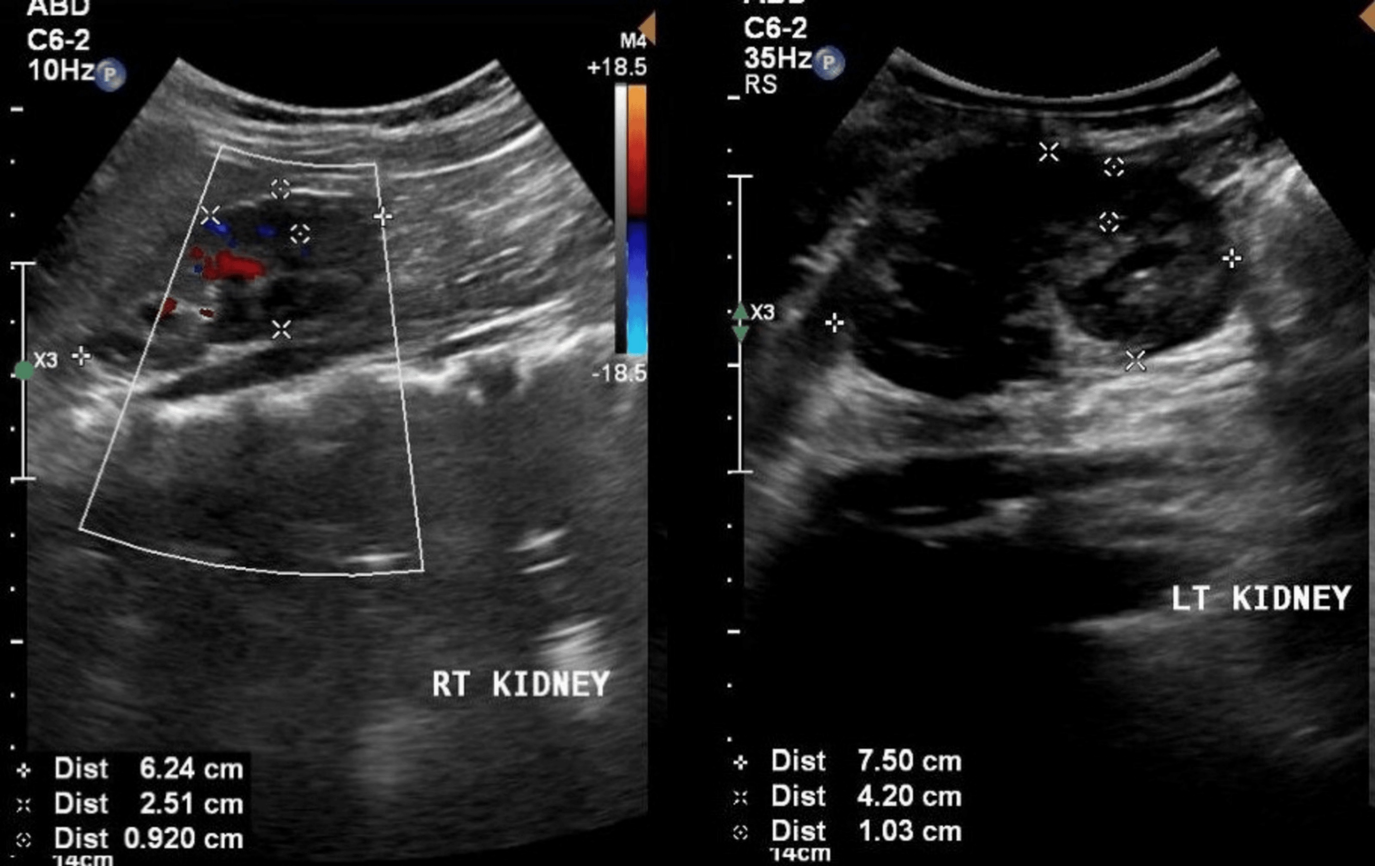
Ultrasound exam of the kidneys, ureters, and bladder (US-KUB) demonstrated improvement in backpressure changes, although mild hydronephrosis persisted.

US-KUB demonstrated improvement in backpressure changes, although mild hydronephrosis persisted (Figure 5).

Long-term follow-up was advised to monitor renal function and bladder compliance, along with close surveillance for urinary tract infections (UTIs), particularly given emerging evidence linking persistent bladder dysfunction to adverse outcomes. 

## Discussion

PUV remains a significant cause of morbidity in the pediatric population. While early presentations are common, late presentations with subtle symptoms pose critical diagnostic challenges and carry important implications for patient outcomes. Delayed diagnosis of PUV often results in progressive renal damage, bladder dysfunction, and a higher risk of long-term complications, including chronic kidney disease, hypertension, and, in some cases, the need for renal replacement therapy. These consequences highlight the importance of timely recognition and intervention [2].

Strategies for earlier detection must prioritize comprehensive antenatal and postnatal care. Prenatal ultrasonography remains a cornerstone of early detection, especially when findings such as oligohydramnios, bladder distension, or bilateral hydronephrosis are observed. Any degree of antenatal hydronephrosis, even when mild, warrants thorough postnatal follow-up. Postnatal ultrasonography within the first week of life is essential for evaluating the urinary tract in at-risk neonates. In cases of suspected obstruction, VCUG remains the gold standard for diagnosis, while urethrocystoscopy serves to confirm the diagnosis and guide treatment planning [8,9].

Educational initiatives targeted at primary care providers and pediatricians are crucial to raising awareness of the subtle presentations of PUV. Persistent lower urinary tract symptoms, even in the absence of overt findings, should prompt detailed evaluation. In resource-limited settings, improving access to diagnostic modalities, including VCUG and postnatal ultrasonography, is vital to ensuring earlier detection and better outcomes.

The primary treatment for PUV is endoscopic valve ablation, which has proven highly effective in relieving obstruction and preserving renal function. Advancements in surgical techniques, such as the use of cold knife, diathermy, Thulium, or Holmium YAG lasers, have further enhanced outcomes. In this case, Holmium YAG laser ablation was utilized, demonstrating high efficacy with minimal complications [7,9]. Management does not end with valve ablation; addressing residual bladder dysfunction is equally critical. Adjunctive therapies such as alpha-adrenergic blockers and clean intermittent catheterization are increasingly recognized as essential components of long-term care, particularly for patients with ongoing voiding dysfunction. Early and aggressive management of bladder dysfunction following valve ablation is critical to minimizing secondary complications and optimizing renal outcomes.

In conclusion, addressing the challenges of late presentation through earlier detection and comprehensive management is essential to mitigate the morbidity associated with PUV. By improving awareness, access to diagnostic tools, and the timely implementation of appropriate treatment, the burden of late-diagnosed PUV and its complications can be substantially reduced, ultimately enhancing patient outcomes.

## Conclusions

Subtle urinary symptoms in children should prompt thorough evaluation for the possibility of PUVs. Early and timely intervention is crucial to prevent long-term renal and bladder complications. Laser ablation has emerged as a highly effective and minimally invasive treatment modality, offering excellent outcomes with minimal complication rates. Long-term follow-up remains essential to monitor renal function, bladder dynamics, and overall growth and development, ensuring optimal outcomes.
